# Anomalous Affection of the Scrotum

**Published:** 1830-04-01

**Authors:** 


### XXVI.

### ANOMALOUS AFFECTION OF THE SCROTUM.

In Dr. Titley's valuable book on the diseases of the male genitals lately published, we find a brief notice of a certain anomalous affection of the scrotum, observed by Mr. Patrick Maxwell in the Island of Antigua.

*Case.* A gentleman, ætat. 35, who had always enjoyed uninterrupted good health, was suddenly seized in the night with a violent pain in the region of the kidney, fever, vomiting, and purging. His scrotum also swelled much and was very painful. The renal affection yielded to the ordinary treatment, but the swelling of the scrotum defied the doctor. It was as large as a child's head, very much corrugated, and quite red; its cellular structure was distinctly filled with fluid; and the testicle and spermatic cord were distinguished free from disease. By bathing the scrotum in cold water it was very considerably and instantaneously diminished, and in the morning it was nearly the natural size.

It was now determined to make incisions into the scrotum, but instead of limpid water oozing out gradatim, as had been expected, there was a large and continued stream of fluid resembling equal parts of blood and milk intimately mixed together.* The quantity then discharged amounted to upwards of three pints; it first coagulated, and then separated into two parts, a crassamentum evidently blood, and a fluid so exactly resembling milk that it was impossible to distinguish it by the appearance; it had no smell and its taste was a little saltish.

When visited in the evening the patient was as well as usual, and the appearance of the scrotum was the same. The lint being removed from the scarification, the discharge again began in a continued stream, as before, and more than a pint of the same kind of fluid was evacuated. Next morning matters continued the same in every respect; on removing the dressings the discharge of more than a pint of the same fluid again took place; and by the same means a similar discharge was induced, both before dinner and at bed-time. In this manner, for the space of twelve days successively, from three to four pints were drawn off daily. Finding the source of the discharge, like the riches of the kingdom of Prester John, to be inexhaustible, the medical attendants allowed the lint to remain on for twenty-four hours without being removed, and the incision was thus healed. He never found himself the least weakened by so considerable a discharge, but went about as usual.

What became of the fluid after the healing of the incision, we are not informed, but if it went on collecting within the scrotum in the ratio at which it had been let out of it, the gentleman must very shortly have had an appendage, as large as the faith of those who can swallow the story. The patient in fact would appear to have had a perfect land of Canaan in the nether part of his abdomen, for it flowed pretty lustily with milk, though certainly not with honey, as the "stream" was a little *saltish.* When Marco Polo, the celebrated Venetian traveller, accurately described the magnificence of the court of Pekin, and the more than Christian state of a Mongul Emperor, he was thought for many centuries to be taking a traveller's licence. But when Sir John de Mandeville, that prince of liars, asserted that he had witnessed a sea of sand with a river of rocks flowing into it, and added with an oath that it furnished excellent fish, the good folks of the day trusted as implicitly to Sir John, and with as good reason too, as some simple-minded persons do to the Lancet at present. Thus there is no saying in this oddly managed world who are fibbing and who are not, and consequently we should be extremely sorry to assert that we do not believe every syllable of Mr. Maxwell's case. The explanation of the phenomena, we leave to our readers.†

* For what possible reason were scarifications proposed, seeing that the scrotum was reduced to nearly its natural size.—ED.

† This case is absolutely beyond the bounds of credibility.—EDITOR.

